# Malignant Disease of the Spine

**Published:** 1895-02-02

**Authors:** A. H. Tubby

**Affiliations:** Surgeon to the National Orthopædic Hospital; Surgeon to Out-Patients, Evelina Hospital for Sick Children


### Medical Progress and Hospital Clinics.

[*The Editor will be glad to receive offers of co-operation and contributions from members of the profession. All letters should be addressed to* THE EDITOR, THE LODGE, PORCHESTER SQUARE, LONDON, W.]

### MALIGNANT DISEASE OF THE SPINE.

By A. H. TUBBY, M.S.Lond., F.R.C.S.Eng. Surgeon to the National Orthopædic Hospital; Surgeon to Out-Patients, Evelina Hospital for Sick Children.

The forms of malignant new growth are sarcoma and carcinoma; these are rarely primary, but secondary to neoplasms elsewhere.

As instances of primary sarcoma, Judson quotes three cases, which were mistaken for Pott's disease: the first case occurred in a boy, aged four and a-half years; the second in a man, aged thirty-five years; and the third, also in a man, aged forty-two years. In one the spinal curves were normal, in another there was doubtful alteration of the curves, while the remaining case showed a prominent eighth dorsal spine, forming a distinct angle. Gibney[1] mentions a case of sarcoma in a man aged forty, the growth being situated in the fifth and sixth dorsal vertebræ; and Howard Marsh alludes to a case in a girl aged seven years. The form of sarcoma is unusually "large-celled," and Michel[2] has described these under the heading of "tumor myeloides."

Examples of primary carcinoma in the spine are sufficiently rare to be pathological curiosities. The majority of cases of malignant disease in the spine are due to secondary carcinoma, following similar disease in the breast, stomach, pancreas, liver, rectum, &c. Hence, if a patient complains of excruciating pain in the back coming on suddenly, and if he or she be middle-aged, it is always well to examine carefully the sites of primary carcinoma. Howard Marsh, in "Bye-ways in the Study of Disease of the Spine,"[3] quotes a case of secondary carcinoma with angular deformity, following cancer of the breast.

An excellent article on "Malignant Disease of the Spine," by R. W. Amidon, appears in the *New York Medical Journal*, February 26th, 1887.

The chief symptoms are pain and paralysis. The pain is intense and excruciating and out of all proportion to any local signs, but it is nevertheless more accurately limited to the diseased area than in Pott's disease. In the latter local pain is not a prominent sign. The pain of growth is capricious, and it is increased by pressure or motion. It may disappear more or less completely at a later period according to Edes. Such an instance came under my own notice, of which the following are the details: A lady, aged forty-nine, who had been twice operated on by an eminent surgeon for carcinoma of the breast, in whom extensive recurrence had taken place, was seen by me on account of agonising pain in the lower dorsal region. This had lasted for four days, and various remedies had been tried. I injected half a grain of acetate of morphia subcutaneously and the pain soon disappeared, nor at any other time did it trouble the patient. Her death occurred three months afterwards. At the autopsy a large mass of secondary growth was found involving the bodies of the tenth and eleventh dorsal vertebræ.

Paralysis, due to spread of the disease to the meninges or to compression of the cord may ensue. Bradford and Lovett[4] state that the occurrence of œdema from thrombosis in paralysis rather favours the probability of this disease as the cause. The prominence, when it is found is generally more rounded in malignant disease than in Pott's disease. Unfortunately nothing can be done in these cases beyond alleviation of pain and distress.

When the disease is secondary recognition is easy, but when primary it is almost impossible. However, intense pain in the back in a middle-aged or old person, unrelieved by rest and ordinary treatment and requiring powerful anodynes to overcome it, is very suggestive of malignant disease of the spine.

[1] Trans. Amer. Orth. Association, vol. IV. [2] Nouv. Dict. de Med. and Chirur., 39, p. 222. [3] Lancet, 1893, vol. II., p. 792. [4] Op. cit., p. 200.

